# Potential Salivary mRNA Biomarkers for Early Detection of Oral Cancer

**DOI:** 10.3390/jcm9010243

**Published:** 2020-01-16

**Authors:** Su Young Oh, Sung-Min Kang, Soo Hyun Kang, Heon-Jin Lee, Tae-Geon Kwon, Jin-Wook Kim, Sung-Tak Lee, So-Young Choi, Su-Hyung Hong

**Affiliations:** 1 Department of Microbiology and Immunology, School of Dentistry, Kyungpook National University, Daegu 700-412, Korea; oohsuy@knu.ac.kr (S.Y.O.); dkdkdk43@knu.ac.kr (S.-M.K.); black_bean@knu.ac.kr (S.H.K.); heonlee@knu.ac.kr (H.-J.L.); 2 Department of Oral and Maxillofacial Surgery, School of Dentistry, Kyungpook National University, Daegu 700-412, Korea; kwondk@knu.ac.kr (T.-G.K.); vocaleo@knu.ac.kr (J.-W.K.); st0907@knu.ac.kr (S.-T.L.)

**Keywords:** oral squamous cell carcinoma, saliva, early diagnosis, mRNA, area under the curve

## Abstract

We evaluated potential biomarkers in human whole saliva for the early diagnosis of oral squamous cell carcinoma (OSCC). We selected 30 candidate genes with relevance to cancer from recent reports in PubMed. Saliva samples were obtained from 34 non-tumor control and 33 OSCC patients. Real-time PCR was performed, and mRNA levels were compared. Normalized mRNA levels of six genes (NGFI-A binding protein 2 (NAB2), cytochrome P450, family 27, subfamily A, polypeptide 1 (CYP27A1), nuclear pore complex interacting protein family, member B4 (NPIPB4), monoamine oxidase B (MAOB), sialic acid acetyltransferase (SIAE), and collagen, type III, alpha 1 (COL3A1)) were significantly lower in saliva of OSCC patients. Receiver operating characteristics (ROC) analysis was used to individually evaluate the predictive power of the potential biomarkers for OSCC diagnosis. The area under the curve (AUC) values were evaluated for the OSCC vs. non-tumor groups via univariate ROC analyses, as well as multivariate ROC analyses of combinations of multiple potential biomarkers. The combination of CYP27A1 + SIAE showed a favorable AUC value of 0.84. When we divided saliva samples into two groups according to age using a 60-year cut-off, with OSCC patients and controls evaluated together, the AUC of MAOB–NAB2 was more predictive of OSCC in the under-60 group (AUC, 0.91; sensitivity, 0.92; and specificity, 0.86) than any other gene combination. These results are expected to aid the early diagnosis of OSCC, especially in patients under 60 years of age. While more studies with larger numbers of patients are necessary, our result suggest that salivary mRNA would be a potent biomarker for early OSCC diagnosis.

## 1. Introduction

Despite the many advances in cancer treatment, the five-year survival rate for patients with oral squamous cell carcinoma (OSCC) has improved only marginally. Therefore, there is critical need to identify markers for the early diagnosis of OSCC. Biomarkers are molecular signatures and indicators of normal biological and pathological process, and thus may provide useful information for the detection, diagnosis, and prognosis of disease. Many studies have attempted to identify cancer biomarkers in non-invasive samples [1,2,3,4]. Saliva has direct contact with OSCC lesions, which gives it particular potential as a specific and sensitive screening tool. While more than 100 potential salivary biomarkers (DNA, RNA, mRNA, miRNA, and protein) have been previously identified for OSCC (reviewed in [5,6,7,8]), further research is required to validate biomarkers for clinical applications.

A previous review identified several possibilities regarding the confounding influence of oral disease (e.g., inflammation, ulcers, and periodontitis) at the time of saliva collection on the correlation of salivary markers for oral cancer [9]. First, it is possible that controlling for potential external confounders is important for these analyses, or perhaps oral conditions do not affect expression of saliva markers. Previous study showed that chronic periodontitis can affect the levels of potential oral cancer salivary mRNA biomarkers [10,11]. In addition, chronic periodontitis is a risk indicator for oral cavity and/or oropharyngeal cancer [12,13]. Specifically, Moraes reported that 89% of oral cancer patients presented severe chronic periodontitis. The association between periodontitis, and carcinogenesis has been explained by chronic inflammation, presence of bacteria and a reservoir for possible carcinogenic agents [14]. A previous study showed no significant differences in biomarker levels as a result of cigarette smoking [15]. 

In clinical medicine, the use of multiple biomarkers in combination can improve diagnostic accuracy [16]. Receiver operating characteristic (ROC) curve analysis is an extremely useful method for evaluating the predictive utility of biomarkers or diagnostic tests [17,18]. For markers with continuously distributed data, the ROC curve graphically depicts the diagnostic ability of the marker for all threshold values in a unit square by plotting the proportion of true positives (sensitivity) versus the proportion of false positives (1 - specificity) [19]. Therefore, we compared the area under the ROC curve (AUC) values for OSCC patients vs. the non-tumor group using univariate ROC analyses and multivariate ROC analyses of combinations of two candidate biomarkers. In the present study, we investigated the potential biomarkers in human whole saliva for the early diagnosis of OSCC. Furthermore, we tried to evaluate the association between subjects’ age and salivary mRNA biomarker levels to increase accuracy.

## 2. Materials and Methods

### 2.1. Patients and Saliva Collection

All participants were recruited from the Department of Oral and Maxillofacial Surgery of Kyungpook National University Dental Hospital from 2015 to 2017. Informed consent was obtained from all patients before the collection of saliva prior to any treatment. All experimental procedures were conducted in accordance with the Declaration of Helsinki and approved by the Ethics Committee of the Kyungpook National University Hospital (KNUH201401021). The study population consisted of two groups: Group 1 included non-OSCC patients (*n* = 34) as controls, and Group 2 comprised OSCC patients (*n* = 33). The control subjects included 13 males and 21 females. The OSCC patient group comprised 22 males and 11 females. Most participants in the control group are patients who visited the hospital for third molars extraction or prosthetic treatment operation. None of the control group had precancerous lesions such as leukoplakia. The major symptoms of OSCC patients were pain, edema, or ulcer, as well as leukoplakia, inflammation, or healing delay. All of them were diagnosed with SCC by incisional biopsy. No patients had suspected symptoms of lichen planus. We included non-tumor control subjects and OSCC patients with mild periodontitis. Mild periodontitis was determined by the amount in clinical attachment loss by records in the Electronic Medical Record database program. In addition, radiographic bone loss was also referred to analyzing radiographic images. The maximum probing depth of mild periodontitis was less than 4 mm and mostly horizontal bone loss [20]. More detailed information of the subjects who participated in this study is shown in Table 1.

Saliva was collected from all of the patients who agreed to participate in this study on their first visit to the hospital. Patients were excluded or included in the non-tumor group according to the diagnosis result. Unstimulated whole saliva samples were collected by previously described methods [10]. Briefly, subjects were requested not to eat, drink, or perform any kind of oral hygiene procedures prior to saliva collection for 1 h. Just before saliva collection, subjects performed oral rinse with a cup of water. Five minutes later, participants were asked to spit into a 50 mL sterile plastic tube. A maximum of 3 mL of saliva was collected within 10 min. After the addition of 3 μL RNase inhibitor, the saliva samples were centrifuged at 5600 rpm, 4 °C for 15 min. The supernatant was transferred to two Eppendorf tubes in 500 μL aliquots and stored at −80 °C until further use. 

### 2.2. Total RNA Extraction

Five hundred microliters of centrifuged saliva was mixed with the same volume of TRIzol solution, followed by incubation for 5 min at room temperature. Then, 200 μL of 1-bromo-2 chloropropane was added, and the contents were mixed by inversion, followed by incubation for 3 min at room temperature. After centrifugation at 12,000 rpm, 4 °C for 15 min, 800 μL of the aqueous phase was transferred into two new Eppendorf tubes in 400 μL aliquots. Then, 800 μL of isopropanol was added, vortexed, and incubated at −20 °C overnight. The next day, the tubes were incubated at room temperature for 10 min, followed by centrifugation at 13,000 rpm, 4 °C for 20 min. The supernatant was discarded, and the pellet was washed with 1 mL of 75% ethanol and centrifuged at 13,000 rpm, 4 °C for 5 min. The RNA pellet was dried at room temperature for 10 min and solubilized with 20 μL of diethyl pyrocarbonate (DEPC) water. The isolated RNA was treated with RNase-free DNase (Ambion Thermo Fisher Scientific, Waltham, MA, USA) according to the manufacturer’s instructions. RNA concentrations were measured using a NanoDrop (Thermo Fisher Scientific, Waltham, MA, USA).

### 2.3. Primary Candidate Genes and Real-Time PCR (qPCR) 

Most previous studies on saliva or serum have explored molecular markers using DNA microarrays. Importantly, substantial differences arise when evaluating mRNA expression of the candidate biomarkers using DNA microarray compared to qPCR. It is currently accepted that the qPCR method has a greater dynamic range and sensitivity than microarray methods [21,22]. Therefore, we selected 30 candidate genes with relevance to cancer according to recent reports in PubMed. Genes that have recently been identified as important in carcinogenesis were also included. Then, mRNA levels of the candidate genes were analyzed by qPCR in saliva samples from non-tumor controls and tumor patients. A list of 30 mRNAs, references, and primer sequences are shown in Table S1.

A total of 500 ng saliva RNA was reverse-transcribed into cDNA using a first-strand cDNA synthesis kit (CELLSCRIPT, LLC. Madison, WI. USA), followed by qPCR. SYBR green PCR master mix was obtained from Enzynomics, Inc. (Daejeon, Korea). All the salivary mRNA samples contained transcripts for GAPDH, which has been used previously as a quality control [23,24]. Therefore, we used GAPDH mRNA level as an internal control. qPCR was carried out using an ABI 7500 real-time PCR system (Applied Biosystems, Foster City, CA, USA) in triplicate. Calculations were performed based on Δcycle threshold (ΔCt) values, which were determined by normalizing the average Ct value of each sample to that of the endogenous *GAPDH* control and then calculating the 2^−ΔΔCt^ value for each treatment.

### 2.4. Comparison with NCI GEO Datasets

To evaluate the expression patterns of candidate biomarkers in OSCC tumor tissues and non-tumor control tissues, we examined publicly available gene expression datasets from NCBI GEO databases. The accession numbers were GSE13601, GSE30784, and GSE37991, respectively [25]. The GSE13601 dataset contains data for tumor and matched normal controls from oral tongue SCC patients (*n* = 20). The GSE 30,784 contains data for OSCC (*n* = 167) and normal oral tissues (*n* = 45). The GSE37991 contains tumor and non-tumor pair-wise sample data from 40 male OSCC patients. The microarray data of GSE13601 and GSE30784 were based on GPL8300 (Affymetrix Human Genome U95 Version 2 Array, Affymetrix Inc., Santa Clara, CA, USA) and GPL570 (Affymetrix Human Genome U133 Plus 2.0 Array), respectively. The microarray data of GSE37991 were based on GPL6883 (Illumina HumanRef-8 v3.0 expression beadchip, Illumina Inc., San Diego, CA, USA).

### 2.5. Statistical Analysis

Subjects’ age was described by mean values and standard deviations. One-way ANOVA for continuous and chi-square test for non-continuous variables were used to check the distribution between two groups. To evaluate the difference of mRNA level between control and OSCC group, Shapiro–Wilk normality test was performed. Both groups showed non-normal distribution for salivary mRNA level. Therefore, the difference of ΔΔCt of each mRNA between the two groups was evaluated using unpaired two-sample Wilcoxon test. The correlation between saliva mRNA levels and OSCC status was calculated using the logistic regression test to yield a linear combination of markers with the ability to discriminate non-tumor subjects from OSCC patents. The area under the ROC curve was used to evaluate the diagnostic accuracy of each mRNA as a potential biomarker for OSCC [26]. The algorithm for the combined analysis of candidate biomarkers is summarized as follows: calculate the empirical AUC value of the ROC curve of each mRNA; order the empirical AUC from largest to smallest; combine the first two candidate mRNAs [27]; create a set of combined mRNAs based on the risk coefficients in the aforementioned step; combine it with the next biomarker; and follow the aforementioned two steps until all candidate biomarker RNAs are included. These data were calculated using R software. *p* values ≤ 0.05 were considered statistically significant. All statistical analyses were performed using the R program (R.3.6.1., The R Foundation, Vienna, Austria).

## 3. Results

### 3.1. Candidate mRNA Levels in Saliva from Non-Tumor Control and OSCC Groups

**Figure 1:**
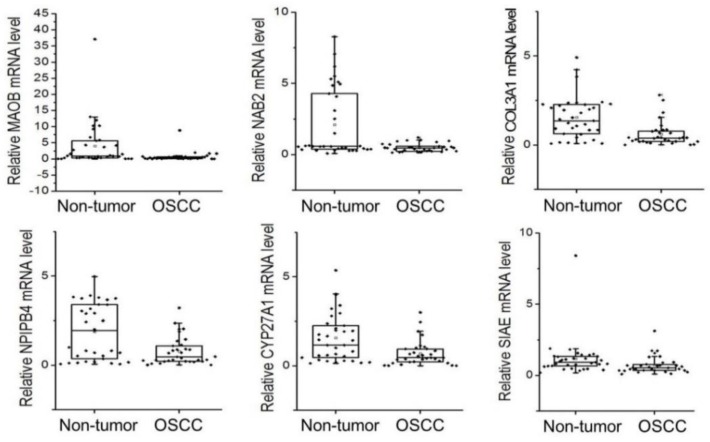
Comparison of salivary mRNAs between control and oral squamous cell carcinoma (OSCC) patients with the relative level (fold change) of the six candidate genes by qPCR analysis. Boxes extend from the third quartile to first quartile, with the line at the median. OSCC: oral squamous cell carcinoma.

**Figure 2:**
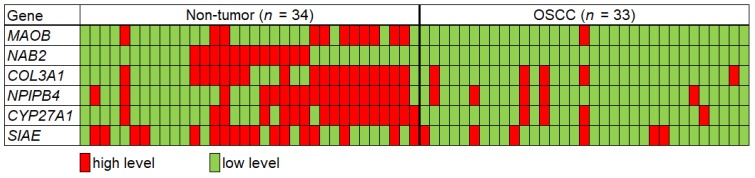
Direct comparison of each mRNA level in the two groups to predict oral squamous cell carcinoma (OSCC). The subjects representing the ΔΔCt below non-tumor group’s average ΔΔCt−SD are marked as “low level” mRNA, which is represented as green color (cancer). The subjects who showed greater ΔΔCt than this value were marked as “high level” expression, which is represented with red color (control). The cut-off of ΔΔCt for cancer diagnosis of each mRNA is 2.80 for MAOB, 1.69 for NAB2, 1.35 for COL3A1, 1.62 for NPIPB4, 1.34 for CYP27A1, and 0.94 for SIAE.

On average, 75.2 ± 20.3 ng (*n* = 57) of total RNA was obtained from 500 μL of saliva supernatant. There was no significant difference in total RNA quantity or quality between the OSCC patients and non-tumor controls. Among the 30 saliva transcripts tested in the present study, six targets, namely *MAOB* (monoamine oxidase B), *NAB2* (NGFI-A binding protein 2), *COL3A1* (collagen, type III, alpha 1), *CYP27A1* (cytochrome P450, family 27, subfamily A, polypeptide 1), *NPIPB4* (nuclear pore complex interacting protein family, member B4), and *SIAE* (sialic acid acetyltransferase), were significantly decreased in the OSCC group (Figure 1 and Table 2). Among the six candidate genes, *MAOB* (fold change = 0.18) and *NAB2* (fold change = 0.23) showed the most reduced mRNA level in the OSCC group compared to control subjects. To predict OSCC with the ΔΔCt value of each candidate gene, the subjects showing each mRNA level below the non-tumor group’s average ΔΔCt −SD were marked as “low level” mRNA (cancer), which is represented as green color in Figure 2. On the contrary, the subjects who showed greater ΔΔCt than this value (control) were marked as “high level” expression, which is marked with red color in Figure 2.

### 3.2. AUC Analysis with Individual or Combinations of Candidate mRNAs

The AUC values from the ROC curves were analyzed for the six candidate mRNAs showing significant differences between OSCC patients and controls. The detailed statistics of the AUCs from ROC curves, sensitivities, and specificities are listed in Table 3. In the univariate ROC analysis, SIAE (AUC, 0.70; sensitivity, 0.79; and specificity, 0.50) mRNA performed most favorably for predicting OSCC among the six potential biomarkers. The AUC values of the six candidate transcripts ranged from 0.63 to 0.70. To improve diagnostic accuracy, we performed combination analysis of the candidate markers. As shown in Table 3, the CYP27A1 + SIAE combination was superior to any other gene sets (AUC, 0.84; sensitivity, 0.73; and specificity, 0.80). MAOB+NAB2 also showed a favorable result (AUC, 0.80; sensitivity, 0.97; and specificity, 0.62).

### 3.3. AUC Analysis of Subjects under 60 Years of Age in Both Groups

**Figure 3:**
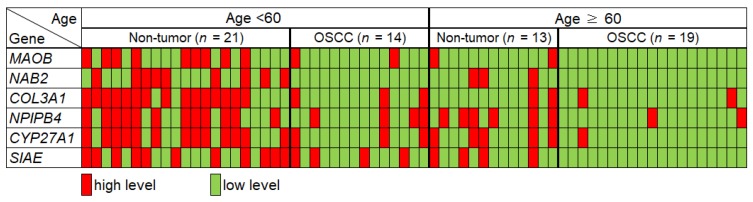
Direct comparison of candidate mRNA levels according to age in the two groups together. The subjects representing the ΔΔCt below non-tumor group’s average ΔΔCt−S.D. are marked as “low level” mRNA, which is represented as green color. The subjects who showed higher ΔΔCt than this value were marked as “high level” expression, which is marked with red color. The cut-off of ΔΔCt for cancer diagnosis (low level) of each mRNA under-60 group is 1.66 for MAOB, 1.65 for NAB2, 1.02 for COL3A1, 1.27 for NPIPB4, 1.22 for CYP27A1, and 0.93 for SIAE.

We separated the patients into those above 60 years of age and those below to obtain a similar number of samples in each group. Average mRNA level of candidate genes and their cut-off values for cancer diagnosis are shown in Table S2. Figure 3 shows salivary transcript levels of subjects under 60 from both groups. The subjects showing expression of each mRNA below the cut-off from non-tumor group’s average ΔΔCt−S.D. were marked as “low level” mRNA (cancer). which is represented as green color in Figure 3. On the contrary, the subjects who showed greater ΔΔCt than this value (control) were marked as “high level” expression, which is marked with red color in Figure 3.

As shown in Table 4, all six potential biomarkers showed remarkably more favorable AUC values in the under–60 group compared to the patients over 60 years of age. In particular, *MAOB*, *COL3A1*, and *CYP27A1* showed remarkably increased AUC values in the under–60 age group (Table 4). When multivariate analysis was performed on the candidate genes in the under-60 group, *MAOB + NAB2* showed an AUC of 0.91 with a sensitivity of 0.92 and specificity of 0.86, which was the most favorable set among the combination data.

### 3.4. Expression Profiles of the Candidate Biomarkers in Tumor Tissue Datasets

**Figure 4:**
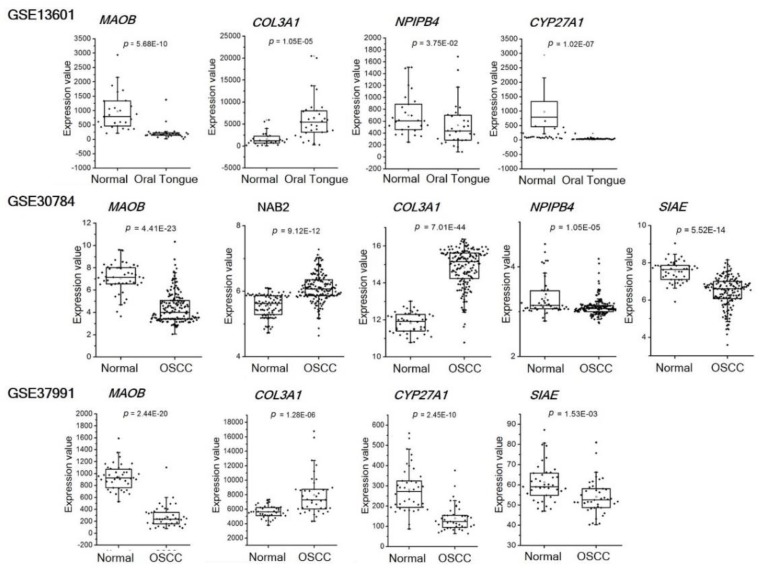
Expression profiles of the candidate mRNAs in OSCC tumor tissues. The expression patterns of the candidate genes in OSCC tumor tissues and non-tumor control tissues were extracted from gene expression datasets of three independent microarray databases (GSE13601, GSE30784, and GSE37991) at NCBI GEO website. The GSE13601 dataset contains data for tumor and matched normal controls from oral tongue SCC patients (*n* = 20). The GSE 30,784 contains data for OSCC (*n* = 167) and normal oral tissues (*n* = 45). The GSE37991 contains tumor and non-tumor pair-wise sample data from 40 male OSCC patients.

Figure 4 shows the significant expression differences (adjusted *p* value < 0.05) between OSCC and normal oral tissues for each GSE dataset. MAOB, NPIPB4, CYP27A1, and SIAE showed significantly lower expression levels in OSCC tumor tissues compared to normal tissues. However, the expression of NAB2 was higher in OSCC tissues in the GSE30784 dataset. COL3A1 also showed a significantly higher mRNA expression level in OSCC tissues in the three datasets analyzed in this study.

## 4. Discussion

Molecular diagnostic markers have been developed for various tumors, and some biomarkers are used clinically. However, OSCC has few available molecular markers for early diagnosis. The use of body fluids such as saliva or serum has shown considerable promise for the early diagnosis of cancer [28,29], but there are few data indicating which salivary transcripts or proteins are up- or down-regulated in cancer patients for use as early diagnostic or prognostic biomarkers. Most of the transcript biomarkers found by microarray are expressed at high levels in cancer patients, while the decreased transcripts have not been actively studied. This study provides novelty by showing that mRNA levels of *MAOB*, *NAB2*, *COL3A1*, *NPIPB4*, *CYP27A1*, and *SIAE* were significantly reduced in the saliva of oral cancer patients. When designing the diagnosis kit for disease, positive or negative control is critical for precise decision. We can anticipate genes of high expression only with negative control, in particular, but positive control plays an important role when it comes to genes that showed decreased expression. Thus, we expect greater difficulty in accurate diagnosis when using biomarkers with decreased expression. Nonetheless, with advanced technology in diagnosis kit development, we expect better use of genes with decreased expression in cancer diagnosis. 

Three datasets from the NCI GEO profile database obtained from OSCC tumor tissues were analyzed to compare with our mRNA expression results in OSCC and normal tissues. Although the mRNA sample sources were different from our study, we thought it might be possible to obtain relevant gene expression information in OSCC patients. Interestingly, the mRNA levels of *MAOB*, *NABNPIPB4*, *CYP27A1*, and *SIAE* were lower in OSCC tumor tissues as compared to normal tissues, which is consistent with our results in the saliva samples. However, *NAB2* showed a significantly higher mRNA level in OSCC tissues compared to normal tissues in the GSE30784 dataset. Salivary mRNA of *COL3A1* also showed an opposite expression pattern in OSCC tissues (GSE13601 and GSE37991). A previous study has shown that mRNA expression trends can vary between tissue RNA and plasma circulating RNA [30]. Because the potential differences in mRNA expression between tumor tissues and saliva cannot be easily deduced, further study is needed. 

In the present study, as the age of the control group and patients decreased, the accuracy of some candidate mRNAs as a biomarker tended to increase. Therefore, the subjects were divided based on the age of 60, which is close to the mean age of the two groups, to evaluate the AUC pattern of candidate mRNAs as OSCC biomarkers according to their ages. Interestingly, salivary *COL3A1* mRNA pattern showed no difference between OSCC patients and control groups in patients over 60 years of age (Figure 3). Overall, the AUC values for individual genes and double gene combinations were more favorable in the under–60 age group than the over–60 group (Table 4). These findings suggest the importance of using specific mRNA biomarkers according to patient age in order to diagnose cancer using patient saliva. A previous study showed that the methylation levels of some genes decreased with age and were lower in cancer patients compared with the normal group. Therefore, these genes were considered potential cancer risk markers that were influenced by age in the process of carcinogenesis [31].

*MAOB* (monoamine oxidase B) is an enzyme located in the mitochondrial outer membrane. It catalyzes the oxidative deamination of biogenic and xenobiotic amines and plays an important role in the metabolism of neuroactive and vasoactive amines in the central nervous system and peripheral tissues [32]. Sharpe et al. reported that *MAOB* was highly expressed in human gliomas [33]. 

However, Chen et al. presented data showing that the protein expression of *MAOA* was significantly decreased in oral cancer tissues compared with adjacent noncancerous tissues [34], which corroborates our data. *NAB2* (NGFI-A binding protein 2) repress the transcriptional activation mediated by early growth response 1. The human *NAB2* gene is located on chromosome 12q13.3–14.1, a region that shows rearrangements in several tumor types. Our previous study showed that NAB2-overexpressing CAFs promote HNSCC progression [35]. However, Abdulkadir et al. showed that NAB2 protein expression is lost in a majority of primary prostate carcinoma specimens early in the tumorigenic process [36]. The expression of *COL3A1* (collagen, type III, alpha 1) in CAFs is known to predict poor prognosis of HNSCC [37]. High *COL3A1* levels were associated with a poor prognosis in bladder cancer [38]. Yuan et al. suggested that *COL3A1* is associated with breast cancer progression by regulating the MAPK signaling pathway. [38] However, the human protein atlas website showed that *COL3A1* is not prognostic in head and neck cancer (ENSG00000168542COL3A1). *CYP27A1* (cytochrome P450, family 27, subfamily A, polypeptide 1) is a vitamin D-regulated enzyme that converts cholesterol to 27-hydroxycholesterol, potentially lowering intracellular cholesterol levels. High *CYP27A1* expression is known to be prognostic for recurrence-free and overall survival [39]. *NPIPB4* (nuclear pore complex interacting protein family, member B4) is a ubiquitously expressed gene in various tissues. The function of this family is unknown, but it shows good conservation from African apes to humans. *SIAE* (sialic acid acetyl esterase) removes acetyl moieties from the hydroxyl groups of sialic acid. Mutations in this gene are associated with susceptibility to autoimmune disease [40]. There have been no data showing a direct relationship between *SIAE* and carcinogenesis. However, higher expression of *O*-acetylated sialic acids has been observed in childhood ALL [41], skin melanoma [42], and human breast cancer [43], suggesting the association of *SIAE* and tumor suppression.

There is still much debate about the limitations of non-tumor controls in the development of biomarkers for early diagnosis of cancer. For OSCC diagnosis, the factors that appeared most commonly in relevant studies include periodontitis, alcohol drinking, and smoking in the control group. Previous study showed that chronic periodontitis can affect the levels of potential oral cancer salivary mRNA biomarkers [10,11]. These data suggest that chronic periodontitis may reduce the accuracy of the diagnosis of oral cancer. As shown in the Table 1, however, since fewer than 10% of all control subjects had mild periodontitis, we anticipate no significant influence on salivary biomarker level in our study. To determine whether periodontitis, smoking, or drinking in the control group affect biomarker levels, we divided the control subjects into a group with one or more of these three factors (*n* = 15) and a group without all three (*n* = 19) and then compared them with the OSCC group. As a result, periodontitis, alcohol drinking, or smoking did not significantly affect the results of this study (data not shown). In addition, the ideal size of control and cancer group for this study was 90–100 at 95% confidence with a 10% sample error, considering the entire Korean population and the OSCC patients who were newly diagnosed during the subjects’ collection period (Korean Statistical Information Service). While more studies with independent cohorts of patients is necessary, our result is valuable for further investigation seeking to improve screening algorithms for the early detection of OSCC. For example, even though general medical history such as diabetes, high blood pressure, and cardiovascular diseases are well-known to have an influence on the salivary mRNA level, the sample size of OSCC patients in our study was too small for such detailed classification. In consideration of the shortcomings, we thus aim to trace the identified biomarkers in future studies and make further investigations on general diseases. In addition, further research is required to evaluate the reliability and validity of these panels for clinical application. More studies involving patients from different ethnic groups may also be necessary to demonstrate the generalizability of these results.

The AUC value is the most popular index used to evaluate overall discrimination accuracy and is commonly used for biomarker evaluation. Higher AUC values indicate superior discriminatory ability of a given diagnostic test or biomarker over all threshold values. Use of biomarker combinations can enhance accuracy, highlighting the importance of developing combinations in diagnostic models [44]. Rather than repeating studies with higher samples sizes performing meta-analyses of multiple studies, ROC analysis is a useful method for evaluating the clinical utility of potentially “promising” biomarkers [45]. While no consensus exists regarding a specific AUC value of ROC curves that represents good predictive power, some publications have recommended an AUC > 0.8 for pursuing the clinical utility of predictive markers, which we support [45]. In addition, our data suggest that salivary mRNA expression may vary according to age independent of disease. 

No previous study has demonstrated the association between age and salivary mRNA level for early cancer diagnosis. In conclusion, our study showed that the two-mRNA panel of C*YP27A1* *+ SIAE* had the highest AUC value (0.84) for early OSCC diagnosis. In particular, *MAOB + NAB2* showed an AUC of 0.91 in the under–60 age group of non-tumor and cancer patients together. Therefore, in the diagnosis of diseases using salivary markers, these data suggest the importance of considering patient age for increased accuracy.

## Figures and Tables

**Table 1:** Subjects participated in this study.

Group		Non-Tumor	OSCC	*p* Value
Number		34	33	
Age	Mean ± SD	53.2 ± 17.4	61.2 ± 18.1	0.007
	Range	25–83	24–97	
Gender	Male	15	22	0.07
	Female	19	11	
Mild periodontitis	Yes	3	10	0.02
	No	31	23	
Smoking	Yes ^1^	9	9	0.94
	No ^2^	25	24	
Alcohol history	Yes ^3^	13	11	0.67
	No	21	22	

^1^ Regular smoking, ^2^ non-smoking or occasional smoking, ^3^ at least once a week. OSCC: oral squamous cell carcinoma.

**Table 2:** Average mRNA levels of six candidate genes in non-tumor controls and oral squamous cell carcinoma (OSCC) groups.

Gene	Non-Tumor (ΔΔCt ± SD)	OSCC (ΔΔCt ± SD)	Relative Fold Change (OSCC/Non-Tumor)	*p* Value	ΔΔCt for Cancer Diagnosis (Non-Tumor−SD)
*MAOB*	4.01 ± 1.20	0.70 ± 0.27	0.18	0.0009	below 2.80
*NAB2*	2.11 ± 0.42	0.49 ± 0.05	0.23	0.0023	below 1.69
*COL3A1*	1.55 ± 0.20	0.62 ± 0.12	0.4	0.0002	below 1.35
*NPIPB4*	1.89 ± 0.26	0.77 ± 0.14	0.41	0.0059	below 1.62
*CYP27A1*	1.57 ± 0.23	0.68 ± 0.12	0.44	0.0016	below 1.34
*SIAE*	1.18 ± 0.24	0.73 ± 0.11	0.62	0.0370	below 0.94

**Table 3:** Sensitivities, specificities, and area under the curve (AUC) values for OSCC samples vs. non-tumor controls.

Gene No.	Gene(s)	Sensitivity	Specificity	AUC	95% CI
One gene	MAOB	0.97	0.35	0.63	0.44–0.82
	NAB2	1	0.35	0.69	0.45–0.95
	COL3A1	0.85	0.53	0.67	0.47–0.79
	NPIPB4	0.85	0.53	0.64	0.47–0.79
	CYP27A1	0.88	0.5	0.64	0.43–0.82
	SIAE	0.79	0.5	0.7	0.56–0.92
Two genes	MAOB + NAB2	0.97	0.62	0.8	0.52–1.0
	MAOB + SIAE	0.82	0.76	0.76	0.63–0.94
	NAB2 + CYP27A1	0.88	0.71	0.81	0.70–0.95
	NAB2 + SIAE	0.82	0.59	0.78	0.62–0.97
	COL3A1 + SIAE	0.7	0.76	0.74	0.59–0.91
	CYP27A1 + SIAE	0.73	0.8	0.84	0.67–1.0

**Table 4:** Age-related Sensitivity, specificity, and AUC values for OSCC vs. control.

Gene No.	Gene (s)	Age < 60			Age ≥ 60		
		Sensitivity	Specificity	AUC	Sensitivity	Specificity	AUC
One gene	*MAOB*	0.92	0.43	0.74	1.0	0.43	0.56
	*NAB2*	1.0	0.43	0.70	1.0	0.23	0.63
	*COL3A1*	0.77	0.71	0.73	0.88	0.15	0.38
	*NPIPB4*	0.69	0.62	0.68	0.94	0.39	0.69
	*CYP27A1*	0.77	0.62	0.73	0.94	0.31	0.56
	*SIAE*	0.70	0.52	0.68	0.94	0.46	0.69
Two genes	*MAOB + NAB2*	0.92	0.86	0.91	1.0	0.39	0.69
	*MAOB + SIAE*	0.70	0.76	0.83	0.88	0.46	0.70
	*NAB2 + CYP27A1*	0.77	0.86	0.88	1.0	0.38	0.69
	*NAB2 + SIAE*	0.69	0.62	0.69	0.88	0.54	0.77
	*COL3A1 + SIAE*	0.54	0.91	0.79	0.82	0.54	0.67
	*CYP27A1 + SIAE*	0.54	0.9	0.82	0.88	0.62	0.72

## Supplementary Materials

The following are available online at https://www.mdpi.com/2077-0383/9/1/243/s1, Table S1: Gene list considered for salivary biomarker, Table S2: Average mRNA level of 6 candidate genes in the under-60 age groups.
