# Supplementary material for: Potential Salivary mRNA Biomarkers for Early Detection of Oral Cancer
Source: J Clin Med. 2020 Jan 16;9(1):243. doi: 10.3390/jcm9010243 (PMC7019677; doi:10.3390/jcm9010243)
Supplement: Supplementary file 1 [file jcm-09-00243-s001.pdf]

Supplementary Table 1. Gene list considered for salivary biomarker.

Symbol	Gene name	Reference	Primer Sequence (5'->3')		Length (bp)	GC%
UBAP1L	Ubiquitin Associated Protein 1 Like	Mazzio EA. et al. [1]	F	GAGGAGCAAGACCTCATTGG	20	55
			R	GAACATCTCCATGGCCTCAT	20	50
GFRA1	Glial Cell Line-Derived Neurotrophic Factor Receptor Alpha1	Esseghir S. et al. [2]	F	TCCAATGTGTCGGGCAATAC	20	50
			R	GGAGGAGCAGCCATTGATTT	20	50
PAPPA	Pregnancy-Associated Plasma Protein A	Torres D. et al. [3]	F	GGCCTCCATCCTACATCTCA	20	55
			R	ATCGCCACAGTACCCACTTC	20	55
RUFY2	RUN And FYVE Domain Containing 2	Lukács J. et al. [4]	F	CTGGTCTGAAGACCCCTCTG	20	60
			R	ACCAGCAGCCCAACAATTAC	20	50
SLC27A5	Solute Carrier Family 27 Member 5	Gao Q. et al. [5]	F	AGCTCCTGCGGTACTTGTGT	20	55
			R	CCTTCTGTGGAGCCGTAGAC	20	60
CREB3L2	CAMP Responsive Element Binding Protein 3 Like 2	Krawczyk KK. et al. [6]	F	ATCCTTCTGCCACCAAGATG	20	50
			R	TGAGCAGGGAGGAACCTCTA	20	55
SIAE	Sialic Acid Acetylesterase	Louwen F. et al. [7]	F	TGCGGCATATCAGTCTGTCC	20	55
			R	CCTAAGTTTTCTGAGGTGGGCT	22	50
ZNF335	Zinc Finger Protein 335	Jen J. et al. [8]	F	GCTTTGCCTCCAAGAACAAG	20	50
			R	TGACTTCCTCCCATCAGGAC	20	55
MMP2	Matrix Metallopeptidase 2	Hsu WH. et. al. [9]	F	GTCCAGAGGCAATGCAGTGGG	21	61.9
			R	TCACTAGGCCAGCTGGTTGGTT C	23	56.5
MMP9	Matrix Metalloproteinase-9	Huang H. [10]	F	CGGTGATTGACGACGCCT	18	61.1
			R	ATACCCGTCTCCGTGCTC	18	61.1
BMP7	Bone Morphogenetic Protein 7	Kang SM. et al. [11]	F	TCGTGGAACATGACAAGGAATT	22	40.9
			R	TGGAAAGATCAAACCGGAACT C	22	45.5
NAB2	NGFI-A Binding Protein 2	Choi SY. et al. [12]	F	CACATCCCTGCTAAAGCTGAA	21	47.6
			R	GTCGAAACGGCCATAGATGAT	21	47.6
NPIPB4	Nuclear Pore Complex Interacting Protein Family Member B4	Duan F. et al. [13]	F	ACCTTTGGGTGTCTCTCCTG	20	55
			R	CCTCAGCCCTTCCTGTCTAC	20	60

Supplementary Table 1. Gene list considered for salivary biomarker-continued

MMP1	Matrix Metallopeptidase 1	Wang QM. et al. [14]	F	AATAGTGGCCCAGTGGTTGA	20	50
			R	GGCTGCTTCATCACCTTCAG	20	55
MAOB	Monoamine Oxidase B	Hodorová I. et al. [15]	F	TCTGCTCTCTGGTTCCTGTG	20	55
			R	GGAGGTCCATTATCCGCTCA	20	55
CYP27A1	Cytochrome P450 Family 27 Subfamily A Member 1	He S. et al. [16]	F	ACGATAAGTTCAGGCAGCAC	20	50
			R	AGGAGGTACATCTCCAGCTC	20	55
Col3A1	Collagen Type III Alpha 1 Chain	Engqvist H. et al. [17]	F	TTGGGATTGCTGGGATCACT	20	50
			R	TGGTTTCCCACTTTCACCCT	20	50
CD44		Chen C. et al. [18]	F	TTTGCATTGCAGTCAACAGTC	21	42.9
			R	GTTACACCCCAATCTTCATGTCCA	24	45.8
DUSP1	Dual Specificity Phosphatase 1	Tuglu MM. [19]	F	CCTACCAGTATTATTCCCGACG	22	50
			R	TTGTGAAGGCAGACACCTACAC	22	50
IL1b	Interleukin 1 Beta	Bent R. et al. [20]	F	CTCTCTCCTTTCAGGGCCAA	20	55
			R	GCGGTTGCTCATCAGAATGT	20	50
S100P	S100 Calcium Binding Protein	Arumugam T. et al. [21]	F	GAGTTCATCGTGTTCGTGGCTG	22	54.6
			R	CTCCAGGGCATCATTTGAGTCC	22	54.6
CYP	Cyclophilin	Lee J. et al. [22]	F	CGGGTCCTGGCATCTTGT	18	61.1
			R	GCAGATGAAAAACTGGGAACCA	22	45.5
CALM1	Calmodulin1	Berchtold MW. et al. [23]	F	TGACAAGGATGGCAATGGTTATA	23	39.13
			R	TACTTCTTCATCTGTTAGTTTTTCTCCT	28	32.1
KLF17	Kruppel Like Factor 17	Shan Zhou S. et al. [24]	F	CTGCCTGAGCGTGGTATGAG	20	60
			R	TCATCCGGGAAGGAGTGAGA	20	55
ATXN-1	Ataxin 1	Kang A. et al. [25]	F	TCCTGCTGTCCGGAGAGAAC	20	60
			R	GCAGACATCCCCAACTGAGAGT	22	54.6
CCL2	C-C Motif Chemokine Ligand 2	Li M. et al. [26]	F	AAGCAGAAGTGGGTTCAGGA	20	50
			R	TGGGTTGTGGAGTGAGTGTT	20	50
ENO1	Enolase-1	Zhu W. et al. [27]	F	CGCCAAGGCCGTGAAC	16	68.8
			R	CGGAGCCAATCTGGTTGACT	20	55

Supplementary Table 1. Gene list considered for salivary biomarker-continued

KPNA4	Karyopherin a-4	Cagatay T. et al. [28]	F	TCAAGCAGTAGTTCAGTCCAATGC	24	45.8
			R	GCTTGCTCACAGACATTCTGATG	23	45.8
NDRG1	N-Myc Downstream Regulated 1	Zhang S. et al. [29]	F	CTCCTGCAAGAGTTTGATGTCC	22	50
			R	TCATGCCGATGTCATGGTAGG	21	52.4
GJA1	Gap Junction Protein Alpha 1	Busby M. et al. [30]	F	CTTCTGGGTCCTGCAGATCA	20	55
			R	ACACCATCAGTTTGGGCAAC	20	50

Supplementary Table 2. Average mRNA level of 6 candidate genes in the under-60 age groups

Gene	Non-tumor (ΔΔCt ± S.D.)	OSCC (ΔΔCt ± S.D.)	Relative fold change (OSCC/ Non-tumor)	P value	ΔΔCt for Cancer diagnosis (Non-tumor - S.D.)
MAOB	2.43 ± 0.77	0.53 ± 0.27	0.22	0.0033	below 1.66
NAB2	2.15 ± 0.49	0.38 ± 0.06	0.18	0.0062	below 1.65
COL3A1	1.28 ± 0.30	0.57 ± 0.16	0.44	0.0046	below 1.02
NPIPB4	1.49 ± 0.22	0.62 ± 0.21	0.42	0.0030	below 1.27
CYP27A1	1.44 ± 0.22	0.69 ± 0.17	0.47	0.0146	below 1.22
SIAE	2.27 ± 1.30	1.07 ± 0.26	0.48	0.3100	below 0.93

References for Table S1

1. Mazzio, E.A.; Soliman, K.F.A. Whole-transcriptomic Profile of SK-MEL-3 Melanoma Cells Treated with the Histone Deacetylase Inhibitor: Trichostatin A. *Cancer Genomics Proteomics*. **2018**, *15*, 349-364, doi:10.21873/cgp.20094.
2. Esseghir, S.; Todd, S.K.; Hunt, T.; Poulsom, R.; Plaza-Menacho, I.; Reis-Filho, J.S.; Isacke, C.M. A role for glial cell derived neurotrophic factor induced expression by inflammatory cytokines and RET/GFR alpha 1 receptor up-regulation in breast cancer. *Cancer Res*. **2007**, *67*, 11732-11741, doi:10.1158/0008-5472.CAN-07-2343.
3. Torres, D.; Hou, X.; Bale, L.; Heinzen, E.P.; Maurer, M.J.; Zanfagnin, V.; Oberg, A.L.; Conover, C.; Weroha, S.J. Overcoming platinum resistance in ovarian cancer by targeting pregnancy-associated plasma protein-A. *PLoS One*. **2019**, *14*, e0224564, doi:10.1371/journal.pone.0224564.
4. Lukacs, J.; Soltesz, B.; Penyige, A.; Nagy, B.; Poka, R. Identification of miR-146a and miR-196a-2 single nucleotide polymorphisms at patients with high-grade serous ovarian cancer. *J Biotechnol*. **2019**, *297*, 54-57, doi:10.1016/j.jbiotec.2019.03.016.
5. Gao, Q.; Zhang, G.; Zheng, Y.; Yang, Y.; Chen, C.; Xia, J.; Liang, L.; Lei, C.; Hu, Y.; Cai, X., et al. SLC27A5 deficiency activates NRF2/TXNRD1 pathway by increased lipid peroxidation in HCC. *Cell Death Differ*. **2019**, doi:10.1038/s41418-019-0399-1.
6. Krawczyk, K.K.; Ekman, M.; Rippe, C.; Grossi, M.; Nilsson, B.O.; Albinsson, S.; Uvelius, B.; Sward, K. Assessing the contribution of thrombospondin-4 induction and ATF6alpha activation to endoplasmic reticulum expansion and phenotypic modulation in bladder outlet obstruction. *Sci Rep*. **2016**, *6*, 32449, doi:10.1038/srep32449.
7. Louwen, F.; Muschol-Steinmetz, C.; Reinhard, J.; Reitter, A.; Yuan, J. A lesson for cancer research: placental microarray gene analysis in preeclampsia. *Oncotarget*. **2012**, *3*, 759-773, doi:10.18632/oncotarget.595.
8. Jen, J.; Wang, Y.C. Zinc finger proteins in cancer progression. *J Biomed Sci*. **2016**, *23*, 53, doi:10.1186/s12929-016-0269-9.
9. Hsu, W.H.; Chiou, H.L.; Lin, C.L.; Kao, S.H.; Lee, H.L.; Liu, C.J.; Hsieh, Y.H. Metastasis-associated protein 2 regulates human hepatocellular carcinoma metastasis progression through modulating p38MAPK/MMP2 pathways. *J Cancer*. **2019**, *10*, 6716-6725, doi:10.7150/jca.35626.
10. Huang, H. Matrix Metalloproteinase-9 (MMP-9) as a Cancer Biomarker and MMP-9 Biosensors: Recent Advances. *Sensors (Basel)*. **2018**, *18*, doi:10.3390/s18103249.
11. Kang, S.M.; Kim, J.; Kang, S.H.; Oh, S.Y.; Lee, H.J.; Kwon, B.M.; Hong, S.H. Up-regulation of Bone Morphogenetic Protein 7 by 2-Hydroxycinnamaldehyde Attenuates HNSCC Cell Invasion. *Anticancer Res*. **2018**, *38*, 5747-5757, doi:10.21873/anticanres.12913.
12. Choi, S.Y.; Oh, S.Y.; Kang, S.H.; Kang, S.M.; Kim, J.; Lee, H.J.; Kwon, T.G.; Kim, J.W.; Hong, S.H. NAB 2-Expressing Cancer-Associated Fibroblast Promotes HNSCC Progression. *Cancers (Basel)*. **2019**, *11*, doi:10.3390/cancers11030388.
13. Duan, F.; Xu, Y. Applying Multivariate Adaptive Splines to Identify Genes With Expressions Varying After Diagnosis in Microarray Experiments. *Cancer Inform*. **2017**, *16*, 1176935117705381, doi:10.1177/1176935117705381.
14. Wang, Q.M.; Lv, L.; Tang, Y.; Zhang, L.; Wang, L.F. MMP-1 is overexpressed in triple-negative breast cancer tissues and the knockdown of MMP-1 expression inhibits tumor cell malignant behaviors in vitro. *Oncol Lett*. **2019**, *17*, 1732-1740, doi:10.3892/ol.2018.9779.
15. Hodorova, I.; Rybarova, S.; Solar, P.; Benicky, M.; Rybar, D.; Kovacova, Z.; Mihalik, J. Monoamine Oxidase B in Renal Cell Carcinoma. *Med Sci Monit*. **2018**, *24*, 5422-5426, doi:10.12659/MSM.909507.
16. He, S.; Ma, L.; Baek, A.E.; Vardanyan, A.; Vembar, V.; Chen, J.J.; Nelson, A.T.; Burdette, J.E.; Nelson, E.R. Host CYP27A1 expression is essential for ovarian cancer progression. *Endocr*

Relat Cancer. **2019**, doi:10.1530/ERC-18-0572.

17. Engqvist, H.; Parris, T.Z.; Kovacs, A.; Nemes, S.; Werner Ronnerman, E.; De Lara, S.; Biermann, J.; Sundfeldt, K.; Karlsson, P.; Helou, K. Immunohistochemical validation of COL3A1, GPR158 and PITHD1 as prognostic biomarkers in early-stage ovarian carcinomas. *BMC Cancer*. **2019**, *19*, 928, doi:10.1186/s12885-019-6084-4.
18. Chen, C.; Zhao, S.; Karnad, A.; Freeman, J.W. The biology and role of CD44 in cancer progression: therapeutic implications. *J Hematol Oncol*. **2018**, *11*, 64, doi:10.1186/s13045-018-0605-5.
19. Tuglu, M.M.; Bostanabad, S.Y.; Ozyon, G.; Dalkilic, B.; Gurdal, H. The role of dualspecificity phosphatase 1 and protein phosphatase 1 in beta2adrenergic receptormediated inhibition of extracellular signal regulated kinase 1/2 in triple negative breast cancer cell lines. *Mol Med Rep*. **2018**, *17*, 2033-2043, doi:10.3892/mmr.2017.8092.
20. Bent, R.; Moll, L.; Grabbe, S.; Bros, M. Interleukin-1 Beta-A Friend or Foe in Malignancies? *Int J Mol Sci*. **2018**, *19*, doi:10.3390/ijms19082155.
21. Arumugam, T.; Logsdon, C.D. S100P: a novel therapeutic target for cancer. *Amino Acids*. **2011**, *41*, 893-899, doi:10.1007/s00726-010-0496-4.
22. Lee, J.; Kim, S.S. Current implications of cyclophilins in human cancers. *J Exp Clin Cancer Res*. **2010**, *29*, 97, doi:10.1186/1756-9966-29-97.
23. Berchtold, M.W.; Villalobo, A. The many faces of calmodulin in cell proliferation, programmed cell death, autophagy, and cancer. *Biochim Biophys Acta*. **2014**, *1843*, 398-435, doi:10.1016/j.bbamcr.2013.10.021.
24. Zhou, S.; Tang, X.; Tang, F. Kruppel-like factor 17, a novel tumor suppressor: its low expression is involved in cancer metastasis. *Tumour Biol*. **2016**, *37*, 1505-1513, doi:10.1007/s13277-015-4588-3.
25. Kang, A.R.; An, H.T.; Ko, J.; Choi, E.J.; Kang, S. Ataxin-1 is involved in tumorigenesis of cervical cancer cells via the EGFR-RAS-MAPK signaling pathway. *Oncotarget*. **2017**, *8*, 94606-94618, doi:10.18632/oncotarget.21814.
26. Li, M.; Knight, D.A.; L, A.S.; Smyth, M.J.; Stewart, T.J. A role for CCL2 in both tumor progression and immunosurveillance. *Oncoimmunology*. **2013**, *2*, e25474, doi:10.4161/onci.25474.
27. Zhu, W.; Li, H.; Yu, Y.; Chen, J.; Chen, X.; Ren, F.; Ren, Z.; Cui, G. Enolase-1 serves as a biomarker of diagnosis and prognosis in hepatocellular carcinoma patients. *Cancer Manag Res*. **2018**, *10*, 5735-5745, doi:10.2147/CMAR.S182183.
28. Cagatay, T.; Chook, Y.M. Karyopherins in cancer. *Curr Opin Cell Biol*. **2018**, *52*, 30-42, doi:10.1016/j.ceb.2018.01.006.
29. Zhang, S.; Yu, C.; Yang, X.; Hong, H.; Lu, J.; Hu, W.; Hao, X.; Li, S.; Aikemu, B.; Yang, G., et al. N-myc downstream-regulated gene 1 inhibits the proliferation of colorectal cancer through emulative antagonizing NEDD4-mediated ubiquitylation of p21. *J Exp Clin Cancer Res*. **2019**, *38*, 490, doi:10.1186/s13046-019-1476-5.
30. Busby, M.; Hallett, M.T.; Plante, I. The Complex Subtype-Dependent Role of Connexin 43 (GJA1) in Breast Cancer. *Int J Mol Sci*. **2018**, *19*, doi:10.3390/ijms19030693.
